# Transcriptome Analysis Reveals the Age-Related Developmental Dynamics Pattern of the *Longissimus Dorsi* Muscle in Ningxiang Pigs

**DOI:** 10.3390/genes14051050

**Published:** 2023-05-08

**Authors:** Sui Liufu, Qun Lan, Xiaolin Liu, Bohe Chen, Xueli Xu, Nini Ai, Xintong Li, Zonggang Yu, Haiming Ma

**Affiliations:** 1 College of Animal Science and Technology, Hunan Agricultural University, Changsha 410128, China; liufusui0816@163.com (S.L.); lanqunstrive@163.com (Q.L.); lxl810711@163.com (X.L.); chenhe0213914@163.com (B.C.); xxl1632501152@163.com (X.X.); 15291270712@163.com (N.A.); p13548614212@163.com (X.L.); study236@163.com (Z.Y.); 2 Guangdong Laboratory for Lingnan Modern Agriculture, Guangzhou 510642, China

**Keywords:** pork, transcriptomics, muscle development, IMF deposition, growing period

## Abstract

The growth and development of the *Longissimus Dorsi* muscle are complex, playing an important role in the determination of pork quality. The study of the *Longissimus Dorsi* muscle at the mRNA level is particularly crucial for finding molecular approaches to improving meat quality in pig breeding. The current study utilized transcriptome technology to explore the regulatory mechanisms of muscle growth and intramuscular fat (IMF) deposition in the *Longissimus Dorsi* muscle at three core developmental stages (natal stage on day 1, growing stage on day 60, and finishing stage on day 210) in Ningxiang pigs. Our results revealed 441 differentially expressed genes (DEGs) in common for day 1 vs. day 60 and day 60 vs. day 210, and GO (Gene Ontology) analysis showed that candidate genes *RIPOR2*, *MEGF10*, *KLHL40*, *PLEC*, *TBX3*, *FBP2,* and *HOMER1* may be closely related to muscle growth and development, while KEGG (Kyoto Encyclopedia of Genes and Genomes) analysis showed that DEGs (*UBC*, *SLC27A5*, *RXRG*, *PRKCQ*, *PRKAG2*, *PPARGC1A*, *PLIN5*, *PLIN4*, *IRS2,* and *CPT1B*) involved the PPAR (Peroxisome Proliferator-Activated Receptor) signaling pathway and adipocytokine signaling pathway, which might play a pivotal role in the regulation of IMF deposition. PPI (Protein-Protein Interaction Networks) analysis found that the *STAT1* gene was the top hub gene. Taken together, our results provide evidence for the molecular mechanisms of growth and development and IMF deposition in *Longissimus Dorsi* muscle to optimize carcass mass.

## 1. Introduction

With the increasing demand for pork quality, how to improve meat quality has become a current hot spot in the research of livestock husbandry. Meat quality is an economically important trait in pigs, including meat color, intramuscular fat content (IMF), marbling, and tenderness [1,2]. Essentially, pork meat quality is closely related to muscle growth and fat content, which are complex traits influenced by environmental, feeding, and slaughter conditions and, for the most part, genetic factors [3,4]. Compared to introduced pigs, Chinese native pigs demonstrate redder meat color, thinner muscle fibers, and higher IMF content [5]. The Ningxiang pig, a typical Chinese local fatty-type pig, is mainly distributed in Hunan province and is known for its bright red meat color, slender fiber, rich and uniform fat distribution between textures, and tender meat quality [6]. Therefore, it is worth investigating the muscle growth and fatness of Ningxiang pigs in different stages.

In recent years, an increasing number of researchers have aimed to explore and elucidate the molecular mechanisms involved in the development of the *Longissimus Dorsi* muscle [7,8]. Comprehensive ATAC-seq and RNA-seq analyses of the *Longissimus Dorsi* muscle in Luchuan and Duroc pigs revealed that the Wnt signaling pathway, the mTOR signaling pathway, and other classical pathways regulated skeletal muscle development and identified some candidate genes (*ASNS*, *CARNS1*, *G0S2*, *PPP1R14C*, and *SH3BP5*) that may be related to muscle development [7]. RNA-sequencing of Debao and Landrace pigs revealed that DEGs were significantly related to metabolic regulation, amino acid metabolism, muscle tissue, and muscle structure development. Meanwhile, key genes *FOS*, *EGR2*, and *IL6* were identified [8]. The current research on the *Longissimus Dorsi* muscle compares the transcriptome results of two pig breeds (e.g., fatty and lean). Few studies focus on the regular age changes in a single pig breed. There is still a need to report on the research on the periodic changes of age mediated in the *Longissimus Dorsi* muscle by Ningxiang pigs. The candidate genes for muscle development are different in different pig breeds.

IMF deposition is mainly dependent on the proliferation, differentiation, and lipid aggregation of adipose precursor cells, and the process of such differentiation is regulated by complex gene transcription [9]. Research on the mechanisms regulating IMF deposition is still limited. What are the molecular mechanisms underlying pork mass traits, especially intramuscular fat formation, and what are the factors involved? Currently, prior work [10] combined the transcriptome and proteome to analyze the *Longissimus Dorsi* muscle tissue of individual Nanyang black pigs with high and low levels of genetic variation and identified 15 genes as candidates for determining genetic differences in fat deposition, among which genes associated with lipid deposition (*ACACA*, *ACSL4,* and *SCD*) and PPARA-centered lipid metabolism regulators (*PPARA* and *UCP3*). As previously reported, Cheng et al. performed whole-transcriptome profiling of individuals with high and low intramuscular fat IMF content, respectively, in the F2 resource population of the Large White Pig × Min Pig, and the results revealed 218 messenger RNAs (mRNAs) with differentially expressed genes mainly involved in cell differentiation, fatty acid synthesis, phylogeny, muscle fiber development, and regulation of lipid metabolism [11]. In addition, a total of 97 DE regulatory RNAs could be identified in quantitative trait locus (QTLs) associated with IMF. The Ningxiang pig is a typical fatty pig breed; however, there is no relevant study on the principles and specific regulatory mechanisms of IMF deposition in the Ningxiang pig.

Pigs, diploids with 18 pairs of autosomes and one pair of sex chromosomes, are highly similar to humans in terms of anatomy, physiology, and genome, making them an ideal animal model for biomedical research [12]. The distinctive features of Ningxiang pigs are excellent muscle quality and high intramuscular fat content [13], which is much higher than exotic breeds, but the research on the excellent germplasm characteristics of Ningxiang pigs is not deep enough, especially the regulatory mechanisms of traits such as muscle growth and IMF, which need to be analyzed. Therefore, in order to investigate the differential genes in the *Longissimus Dorsi* muscle of Ningxiang pigs’ natal stage (day 1), growing stage (day 60), and finishing stage (day 210) of growth periods, the tissues were selected as the study materials to explore the differences and similarities of gene expression in Ningxiang pigs at the mRNA level. The results of the current study could provide a reference for further research on the function and mechanism of mRNA in the *Longissimus Dorsi* muscle of Ningxiang pigs, as well as an understanding of developmental characteristics in actual production.

## 2. Materials and Methods

### 2.1. Animals and Sample Collection

A total of 9 half-sibling Ningxiang male pigs were provided by the Ningxiang original breeding farm of Chuweixiang Agriculture and Animal Husbandry Co. Ltd. (Ningxiang, Hunan, China). A total of 9 healthy half-sibling Ningxiang male piglets of similar body weight were randomly selected, and all experimental pigs were fed nutritional standards and managed in the same manner and under the same conditions from birth. *Longissimus Dorsi* muscle samples were collected at slaughter during the natal stage (day 1), growing stage (day 60), and finishing stage (day 210) of growth periods, and three tails were randomly selected at each stage (*n* = 3). At the growing stage, all pigs were fed the same diet twice a day using piglet feeds containing 18–19% crude protein, lysine 1.2% or more, 0.20–0.40% sodium chloride, 1.8~2% calcium hydrogen phosphate, and 3300–3400 kJ of digestible energy. At the finishing stage, all pigs were fed the same diet twice a day using compound feeds containing 14–16% crude protein, 0.25–0.60% sodium chloride, 0.60–1.50% calcium, 3100–3200 kJ of digestible energy, and ad libitum water. For the collection of *Longissimus Dorsi* muscle samples, all muscle tissue samples were collected from the same part of each animal (*Longissimus Dorsi* muscle of the fourth to last rib) at each collection time point by professional personnel and immediately frozen using liquid nitrogen (−196 °C) for short-term transport. They were then transferred to a −80 °C freezer for RNA extraction. All animal experiments in this study were approved by the Institutional Animal Care and Use Committee of Hunan Agricultural University, Changsha, Hunan Province, China, with approval number CACAHU 20220101.

### 2.2. Hematoxylin and Eosin (HE) Staining

The *Longissimus Dorsi* muscle tissues from the fourth to last rib were collected and fixed in a 4% paraformaldehyde (Biosharp, Hefei, China) solution for more than 24 h. Then the *Longissimus Dorsi* muscle tissues were dehydrated in a Leica ASP200S automatic dehydrator with gradient alcohol. The tissues were embedded in an embedding machine, and 4 μm thick sections were cut with a YD-355AT paraffin sectioning machine and stained with hematoxylin-eosin. Histological analysis was performed on all specimens, and the diameter of the muscle fascicles (μm) was observed under a microscope.

### 2.3. RNA Extraction and Sequencing Analysis

Total RNA from *Longissimus Dorsi* muscle tissues was isolated using the Accurate SteadyPure Universal Genomic RNA Extraction Kit (Accurate Biology, Changsha, China). The 1% agarose gel was used to identify degradation and contamination. Furthermore, RNA integrity and concentration were evaluated by an ultraviolet spectrophotometer (Thermo Scientific, Waltham, MA, USA) and an Agilent 2100 Bioanalyzer (Agilent Technologies, Santa Clara, CA, USA), respectively. After quality evaluation, certified RNA samples were selected for library construction by sequencing on the Illumina NovaSeq 6000 platform (Illumina, San Diego, CA, USA). Subsequently, Trim Galore (version 0.4.1) was undertaken to remove adapter sequences, read lengths lower than or equal to 20, and reads with more than 50% bases having a quality score (Q) less than or equal to 20. The filtered, clean reads were then analyzed by FastQC (version 0.11.7) to obtain a quality control report. The purified reads were compared to the porcine reference genome (Sus scrofa 11.1) using HISAT2 (version 2.0.1).

The expression level of each gene was quantified by fragment per kilobase of transcript per million reads (FPKM) using Cufflinks software (version 0.17.3) based on the length of the genes and the number of read counts mapped to them. DEGs were calculated between the three comparison groups (day 1 vs. day 60, day 60 vs. day 210, and day 1 vs. day 210) using the DESeq2 package. The selection criteria for DEGs followed the following thresholds: |log2(fold change)| ≥ 1 and false discovery rate (FDR) < 0.05. Gene Ontology (GO) annotation analysis was performed utilizing clusterProfiler (version 4.0). The enrichment module of KOBAS (version 3.0) was used to implement the results of the Kyoto Gene and Genome Encyclopedia (KEGG) pathway analysis. The screening thresholds for both GO and KEGG analyses were set to FDR *p* values < 0.05.

### 2.4. Quantitative Real-Time PCR (qRT-PCR) Validation

Tissue RNA was extracted by the conventional trizol (Invitrogen, Carlsbad, CA, USA) method. The muscle samples were cut to 50–100 mg in a 2 mL centrifuge tube, and 1 mL of Trizol was added to the tissue grinder (Shanghai Jinxin Industrial Development Co., Ltd., JXFSTPRP-32L, Shanghai, China) to break the tissues thoroughly in each tube. A quantity of 200 μL of pre-cooled chloroform was added and centrifuged at 12,000 rpm for 15 min. Carefully, 500 μL of the clear layer was aspirated into a 1.5 mL centrifuge tube, and an equal volume of pre-cooled isopropanol was added. The tube was then centrifuged at 12,000 rpm for 10 min. The white precipitate at the bottom of the tube after centrifugation was RNA. The liquid in the tube was carefully poured off, and 1 mL of 75% ethanol was added (prepared with DEPC (diethylpyrocarbonate, Biosharp, Hefei, China) and 100% anhydrous ethanol) that had been prepared in advance and pre-cooled, and centrifuged for 10 min at 4 °C and 7500 rpm. The resulting solution was carefully poured off the ethanol supernatant, and the centrifuge tube was inverted onto absorbent paper to dry. The liquid in the centrifuge tube was left to dry but not completely dry where it contained the RNA precipitate, and DEPC was added according to the size of the precipitate to dissolve the RNA precipitate. Additionally, cDNA was obtained by reverse transcription of RNA with the PrimeScript^TM^RT reagent kit with a gDNA Eraser (TaKaRa, Tokyo, Japan). Real-time quantitative polymerase chain reaction (qRT-PCR) was used to quantify the expression of *RIPOR2*, *MEGF10*, *KLHL40*, *TBX*, *PLIN5*, *PLIN4*, and *PARP9* mRNA expression levels in the *Longissimus Dorsi* muscle tissues. The qRT-PCR analysis was performed using the Applied Biosystems Quant Studio 7 Flex Real-Time PCR System (Thermo Fisher, Waltham, MA, USA). Based on the reference sequence on NCBI, the fluorescent quantitative primers for the above genes and the primers for the *GAPDH* gene as an internal reference were designed using Primer 5.0 (Table 1) and synthesized by Tsingke Biotechnology Co., Ltd (Beijing, China). Using cDNA as a template, a 20 µL reaction system was prepared: PowerUPTM SYBR^TM^ Green Master Mix (Thermo Fisher, USA), 10 µL of both upstream and downstream primers (10 µM), and 0.2 µL each of both ddH_2_O (8.6 µL) and cDNA template (1 µL). Reaction conditions were set: 50 °C pre-denaturation for 2 min, 95 °C for 10 min, 1 cycle; 95 °C for 15 s, 60 °C for 1 min, 40 cycles. Three replicates of each sample were normalized with *GAPDH* as an internal reference gene using the 2^−ΔΔCT^ method.

### 2.5. Statistical Analysis

The statistical analysis for this study was performed using the Student’s *t*-test in the R environment (version R-4.2.1), and the data are shown as mean ± standard error (SEM). Both R and GraphPad Prism 8.0 software were used to visualize the data.

## 3. Results

### 3.1. Morphological Changes of Longissimus Dorsi Muscle Tissues and Overview of Transcriptomics Data

**Figure 1:**
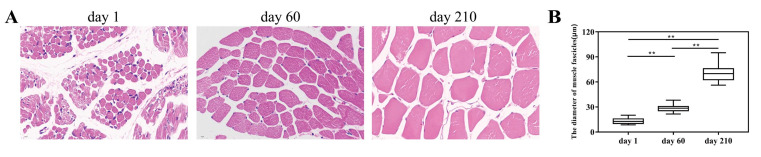
Morphological changes of *Longissimus Dorsi* muscle tissues. (**A**) HE-stained samples of the muscle (630×). (**B**) The comparative analysis of muscle fascicle diameter at three different stages, and ** represents an extremely significant difference (*p* < 0.01).

Upon morphological examination of the *Longissimus Dorsi* muscle in Ningxiang pigs at key periods after birth, it was found that the diameter of the muscle fascicles expressed a progressive increase with age (Figure 1A) and that the diameters of the muscle fascicles at three different time points were statistically significantly different from each other (Figure 1B). Accordingly, we utilized transcriptome sequencing analysis to investigate which genes play a role in the *Longissimus Dorsi* muscle at different developmental stages on days 1, 60, and 210 after birth. In the experiment, nine samples were tested for transcriptome sequencing analysis, and an average of 44.8 million clean reads for Ningxiang pigs were obtained from three stages, and the percentage of Q30 bases was more than 90.52%. After obtaining the clean reads, we used HISAT2 to align these clean reads with the Ningxiang pig reference genome in the range of 95.35–96.03% (Table 2).

### 3.2. Identification of DEGs in the Transcriptome of Longissimus Dorsi Muscle at Three Different Developmental Stages

**Figure 2:**
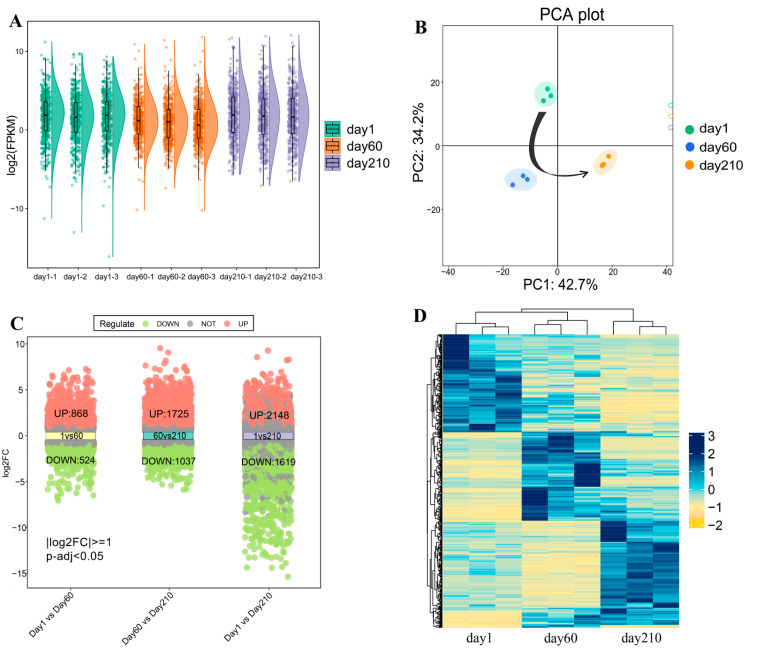
Analysis of differentially expressed genes. (**A**) Boxplot of log2 (FPKM) values for mRNA profiles across different time points. (**B**) PCA diagram of all samples, with the arrow displaying the developmental trajectory. (**C**) The volcano plot shows the number of differentially expressed genes (DEGs), including upregulated and downregulated genes, in three comparison groups (day 1 vs. day 60, day 60 vs. day 210, and day 1 vs. day 210). The threshold for differential expression genes is |log2(FC)| > 1 and an adjusted *p*-value < 0.05. (**D**) Heatmap displaying gene expression patterns.

The boxplot distribution of the log-transformed FPKM revealed the median and quartiles of mRNA expression between the three developmental time points (Figure 2A). Principal component analysis (PCA) demonstrated that different groups could be relatively easily distinguished (Figure 2B), further illustrating the excellent quality of the RNA-seq data. Additionally, differential gene expression analysis was also conducted to achieve DEGs in the different comparison groups. More concretely (Figure 2C), a total of 1392 DEGs (868 upregulated and 524 downregulated) were found in the day 1 vs. day 60 comparison group; 2762 DEGs (1725 upregulated and 1037 downregulated) were identified in the day 60 vs. day 210 comparison group; and 3767 DEGs were implemented in the day 1 vs. day 210 comparison group (2148 upregulated and 1619 downregulated). Ultimately, 441 DEGs were identified from the two comparison groups (day 1 vs. day 60 and day 60 vs. day 210; Table S1). The heat map showed the expression patterns at different stages (Figure 2D).

### 3.3. GO Enrichment Analysis

**Figure 3:**
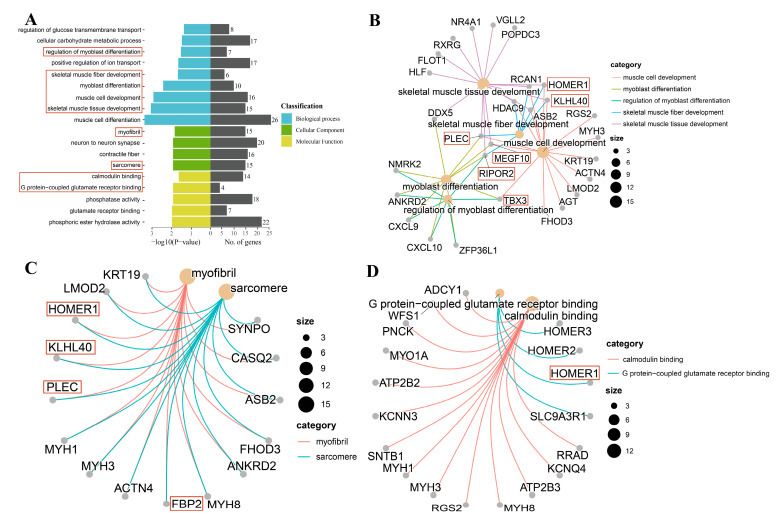
GO analysis of differentially expressed genes (DEGs). (**A**) GO functional classification of differentially expressed genes; (**B**) GO enrichment in biological processes of differentially expressed genes; (**C**) GO enrichment in cellular components of differentially expressed genes; (**D**) GO enrichment in molecular functions of differentially expressed genes.

Based on the targeted DEGs, we performed functional enrichment analysis to obtain GO terms that play a key role in muscle development. In general, GO terms were classified into three major categories: biological processes (BP), molecular functions (MF), and cellular components (CC). As displayed in Figure 3A, the annotated results of BP were mainly related to muscle development, such as muscle cell development, myoblast differentiation, regulation of myoblast differentiation, skeletal muscle fiber development, and skeletal muscle tissue development. Myofibril and sarcomere were enriched in the CC category. In the MF category, calmodulin binding and G protein-coupled glutamate receptor binding were annotated. Specifically, we found that genes *RIPOR2*, *MEGF10*, *KLHL40*, and *TBX3* were co-annotated into at least three BP processes (Figure 3B). *PLEC* and *FBP2* were co-involved in myofibrils and sarcomeres (Figure 3C). *HOMER1* participates in G protein-coupled glutamate receptor binding (Figure 3D). Detailed information on the above genes associated with muscle growth can be found in Table 3.

### 3.4. KEGG Enrichment Analysis

**Figure 4:**
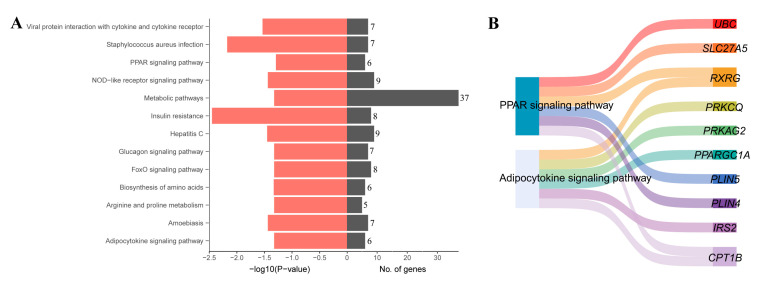
KEGG enrichment analysis of differentially expressed genes (DGEs). (**A**) KEGG enrichment terms. (**B**) DEGs involved in fat-associated pathways.

KEGG analysis of the DEGs revealed that the PPAR signaling pathway and the adipocytokine signaling pathway might play an important role in the development of IMF (Figure 4A). Genes *UBC*, *SLC27A5*, *RXRG*, *PRKCQ*, *PRKAG2*, *PPARGC1A*, *PLIN5*, *PLIN4*, *IRS2*, and *CPT1B* were annotated into these two pathways (Figure 4B).

### 3.5. Network Analysis of Target Genes

**Figure 5:**
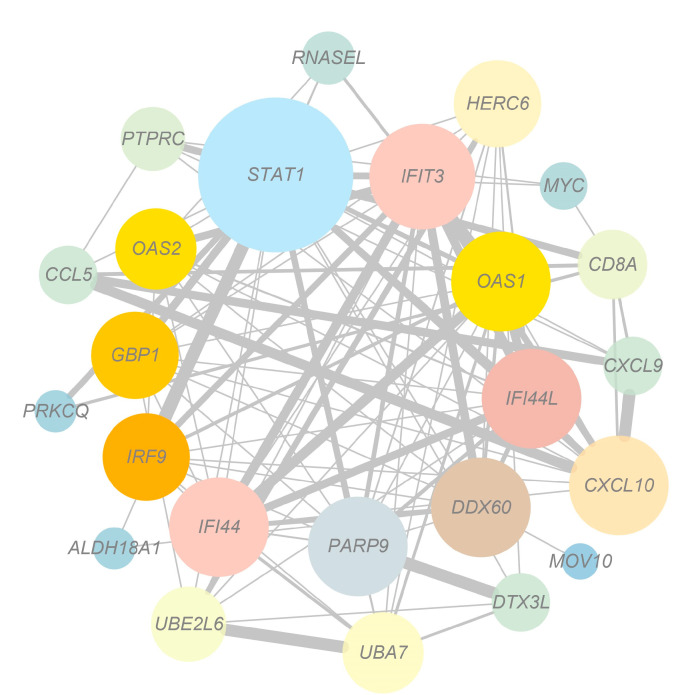
Protein-Protein Interaction Networks.

The DEGs in the regulatory networks were analyzed using STRING to obtain the PPI network (Figure 5). The results revealed that *STAT1* was the top hub gene; and *STAT1*; *IFIT3*; *OAS1*; *IFI44L*; *DDX60*; *PARP9*; *IFI44*; *IRF9*; *GBP1*; and *OAS2* were the ten ranked genes.

### 3.6. Validation of the Results by qRT-pCR

**Figure 6:**
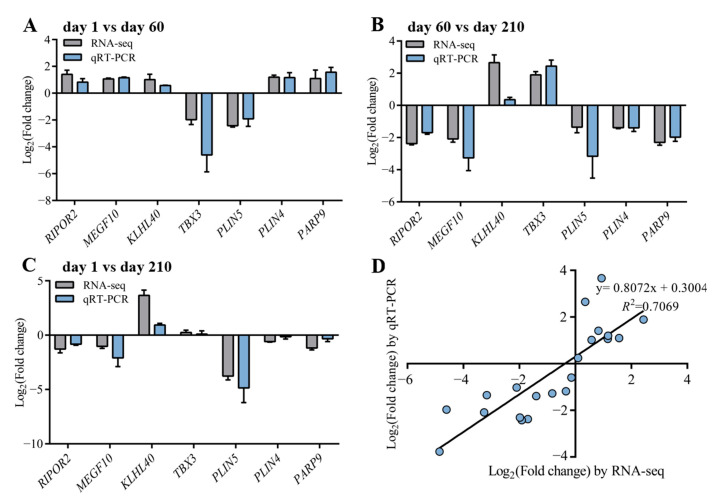
Verification of RNA-seq data by qRT-PCR. (**A**–**C**) Histogram of RNA-seq and qRT-PCR expression levels of day 1 vs. day 60, day 60 vs. day 210, and day 1 vs. day 210, respectively. The *x*-axis represents seven selected genes, and the *y*-axis represents the expression levels of genes from RNA-seq and qRT-PCR. (**D**) The linear regression analysis of expression level between RNA-seq and qRT-PCR data. The *x*-axis represents the log2 fold change of RNA-seq, and the *y*-axis indicates the log_2_ fold change of qRT-PCR.

To verify the accuracy of RNA-seq data, seven genes (*RIPOR2*, *MEGF10*, *KLHL40*, *TBX3*, *PLIN5*, *PLIN4,* and *PARP9*) were randomly selected from the shared DEGs of day 1 vs. day 60 and day 60 vs. day 210 for qRT-PCR analysis. The results showed that the expression patterns of these genes in qRT-PCR were consistent with RNA-seq (Figure 6A–C), and the correlation between the two methods was relatively strong, with a correlation coefficient of 0.8408 (*R*^2^ = 0.7069, Figure 6D), indicating that the DEGs identified from RNA-seq in this study were reliable.

## 4. Discussion

Pork quality is principally dependent on the fat-to-lean mass ratio [23]. Myocytes determine muscle production as well as muscle fiber type, while adipocytes determine fat deposition, and muscle and fat conjointly influence pork quality [24,25]. As a corollary, the interaction between muscle and fat has an important role in the formation of pork quality. As a fatty-type pig, Ningxiang pigs are characterized by excellent meat quality, rich and uniform intertextual fat, and high IMF content [26]. There is insufficient research on the excellent meat quality characteristics of Ningxiang pigs, especially the temporal effect of muscle and IMF development. Therefore, we undertook a transcriptome analysis of the *Longissimus Dorsi* muscle to investigate the underlying biological processes that influence the development of the *Longissimus Dorsi* muscle. The results of our study may contribute to guiding the improvement of pork quality in terms of genetic and molecular aspects and be important for the regulation of muscle growth and development in Ningxiang pigs.

In response to the study, we investigated the morphological differences of the *Longissimus Dorsi* muscle in Ningxiang pigs during the important period of growth and development after birth. We observed that the diameter of muscle fascicles demonstrated a gradual increase with age. Our results confirmed previous observations that there are tremendous changes in the growth performance and histological characteristics of the muscles of mammals as time progresses [27]. Thus, we further performed RNA-seq to reveal the molecular information behind muscle development.

In this research, the results of GO enrichment analysis revealed that some DEGs like *RIPOR2*, *MEGF10*, *KLHL40*, *PLEC*, *TBX3*, *FBP2*, and *HOMER1* might play pivotal roles in the dynamic development of muscle. It was shown that *RIPOR2* (Rho family-interacting cell polarization regulator 2) exerts inhibitory effects on RHO activity and cellular functions affected by RHO, mainly related to RHO signaling and skeletal muscle cell migration, associated with growth and differentiation [28]. Another impressive finding [29] was the intrinsic relationship between *RIPOR2* and T cells, with significantly higher levels of CD^8+^ T cells with high *RIPOR2* expression. Meanwhile, *RIPOR2* has RHOA-dependent effects on various T cell activities, including T cell polarization, adhesion, and migration. Interestingly, *RIPOR2* expression was significantly higher on day 60 than on days 1 and 210 in this study, suggesting that immunological function during the growing stage is crucial for muscle growth and development. *MEGF10* (multiple epidermal growth factor 10) is expressed in muscle satellite cells in skeletal muscle [15,30], and MEGF10 deficiency is associated with impaired muscle regeneration due in part to defects in satellite cell function, causing MEGF10 myopathy [31]. The expression of the *MEGF10* gene was highest during the growing stage, indicating its importance in muscle growth and development. *KLHL40* (kelch-like family member 40) results in a nemaline-like myopathy in mice that closely phenocopies muscle abnormalities observed in KLHL40-deficient patients [32], leading to muscle weakness characteristics as well [33]. *PLEC* (Plectin) is a cell-linked protein that was found to play an important role in promoting C2C12 myoblast differentiation and proliferation but also inhibited their apoptosis, and *PLEC* regulated typical Wnt signaling-mediated skeletal development by downregulating the cellular autophagic degradation system [34]. The genes *KLHL40* and *PLEC* were consistently elevated during three developmentally important stages to promote myoblast proliferation and differentiation.

In addition, *TBX3* (T-box transcription factor 3), an important regulator of muscle and its accessory tissues, exerted an instrumental role in muscle and bone development, and mutations in humans led to Ulnar-mammary syndrome [35]. *FBP2* (Fructose 1,6-bisphosphatase 2), in spite of being a key rate-limiting enzyme for gluconeogenesis, protects mitochondria from calcium stress and plays a key role in the regulation of the cell cycle [20,36]. Downregulation of *FBP2* significantly elevated basal and compensatory glycolysis in adult myoblasts, upregulated mitochondrial respiration, increased the number of mitochondria in cells, and thus improved cellular metabolism [37]. In the finishing stage, *FBP2* was significantly downregulated and was considered a biomarker. *HOMER1* (Homer1 homer scaffolding protein 1), which regulates RyR1 by direct interaction, is an intracellular calcium (Ca^2+^) release channel in the sarcoplasmic reticulum (SR) membrane of the skeletal muscle ryanodine receptor protein, which is required for skeletal muscle excitation-contraction coupling [38]. The molecular function associated with muscle development in our study was found to be calmodulin binding, and in parallel, the gene *HOMER1* was upregulated from the natal stage to the finishing stage in the current study, which is consistent with the result that Ca^2+^ release regulates cell movement, muscle contraction, growth, differentiation, and many other physiological functions.

Furthermore, the KEGG analysis found several fat-associated genes (*RXRG*, *PLIN5*, *PLIN4*, and *CPT1B*) involved in the PPAR signaling pathway and the adipocytokine signaling pathway. With time, we found that the gene *RXRG* showed an upregulation trend. *PLIN5* and *CPT1B* showed opposite tendencies. *RXRG* (Retinoid X Receptor γ) encodes a member of the retinoid X receptor (RXR) family, which is involved in mediating the antiproliferative effects of retinoids (RA). In caponized chickens with low cellular lipid content, RXRG mRNA levels were significantly downregulated [39]. *PLIN5* (Perilipin 5), a scaffolding protein, plays an important role in the formation of lipid droplets [40]. IMF is stored in lipid droplets, and overexpression of *PLIN5* in vitro could significantly reduce the size of lipid droplets, promote the metabolism of triglycerides and the mitochondrial respiratory chain, and enhance thermogenesis. Physiologically, the IMF content in Ningxiang pigs increased over time, and the expression of *PLIN5* exhibited a downregulated trend, suggesting that it might exert an important role in IMF accumulation. While *PLIN4* (Perilipin 4), a coating protein and regulator of lipid droplets, showed an upregulated trend only in the middle stage (day 60). Upregulation of PLIN4 expression resulted in an increase in intracellular lipid droplet deposition, and in addition, inhibition of lipid droplet storage by downregulating *PLIN4* promoted the survival of SH-SY5Y cells [41]. It is speculated that Ningxiang pigs need a large amount of IMF deposition in the early stage (day 1 to day 60), but as IMF deposition increases, *PLIN4* expression needs to be reduced later to mitigate mitochondrial damage and ensure cell survival in the finishing stage [41]. *CPT1B* (Carnitine Palmitoyl transferase 1B), a key rate-limiting enzyme for fatty acid β-oxidation, has an important regulatory role in the catabolic energy supply of fatty acids [42]. It has been shown that fat pigs have higher IMF and lower *CPT1B* gene expression levels compared to lean pigs [42]. The mechanism for the higher IMF content in finishing pigs may be due to a higher capacity for adipogenesis and fatty acid transport but a lower capacity for lipolysis. Consequently, we hypothesized that *CTP1B* played an important role in IMF development and is a potential biomarker for IMF development. These simulations revealed that the PPAR signaling pathway and adipocytokine signaling pathway may be networked with pathways related to lipid metabolism, thus affecting IMF deposition in Ningxiang pigs.

Correspondingly, the PPI interaction network revealed that *STAT1* was the core gene. The *STAT* (signal transducer and activator of transcription) family plays a regulatory role in adipogenesis and is involved in the Jak (Janus kinase)-STAT signaling pathway, which is one of the most characteristic cellular signaling pathways in the immune system [43,44]. *STAT1*, a member of the STAT family, is associated with adipocyte differentiation [45,46], influences lipid metabolic processes, and plays an important role in the process of muscle regeneration [47]. Previous studies have shown that *STAT1* activation enhanced the release of cellular pro-inflammatory cytokines [48], and in injured mice, *STAT1* deficiency promoted accelerated myogenic cell growth [47]. In the current experiment, day 60 was a period of muscle and fat generation in Ningxiang pigs, while the expression of *STAT1* and related immune factors showed a significant upregulation during this period, speculating that many other factors in different microenvironments controlled the function of *STAT1*. The presentation of downregulated *STAT1* on day 210 or the ability to increase immune system response may contribute to the acceleration of muscle and IMF growth, which provides a practical application for our study. Moreover, although we are aware that *STAT1* is an important transcription factor for immune response signaling, it is not clear from our studies how *STAT1* regulates the expression of inflammatory cytokines and chemokines. In the future, it would be useful to extensively elucidate the regulation of these factors by *STAT1*.

The findings in the current study may provide additional insights into specific molecular mechanisms regulating the development of the *Longissimus Dorsi* muscle. With further functional validation, these genes could be used to improve carcass composition. Nevertheless, our work still has some limitations. For instance, we selected only three growth points instead of other important stages, such as pregnancy. Additionally, other histological techniques, such as proteomics and lipidomics, were not implemented in the current study. Therefore, it is of paramount concern that future studies focus on these aspects.

## 5. Conclusions

In our study, a considerable number of DEGs were identified based on transcriptomic analysis of the *Longissimus Dorsi* muscle tissues at different growth stages. According to the annotation results of DEGs in the GO database, functional genes *RIPOR2*, *MEGF10*, *KLHL40*, *PLEC*, *TBX3*, *FBP2,* and *HOMER1* may be strongly related to muscle growth and development. KEGG results revealed that the functional genes *RXRG*, *PLIN5*, *PLIN4*, and *CPT1B* were significantly enriched in lipid metabolism-related pathways of the PPAR signaling pathway and the adipocytokine signaling pathway. PPI network analysis of DEGs in the modulation network demonstrated that *STAT1* was the core gene affecting the growth and development of the *Longissimus Dorsi* muscle and IMF deposition. Taken together, these results facilitate further understanding of the molecular regulatory mechanisms of muscle growth and development and IMF deposition and thus enhance pork quality. In the future, we can conduct joint multi-omics studies at several important stages to improve the quality of pork.

## Figures and Tables

**Table 1:** Primers for real-time fluorescent quantitative PCR.

Gene	Primer Sequence (5′-3′)	Annealing Temperature (°C)	Amplicon Length (bp)	GenBank No
RIPOR2	F: CACGCCTTCTTTTCTAATCTACCG	60	86	XM_047419593.1
	R: CTGAAGCTGAGGGACAGTGG			
MEGF10	F: AATCTCAGGCTCTCGGGTTG	60	139	XM_021084704.1
	R: GGCTGCACACATTAGGGTCT			
KLHL40	F: CCACCAATGTGGTCATACACG	60	107	NM_001165887.2
	R: TGCCCCTGTAGTGTATCCTT			
TBX3	F: CGGACAAACACGGATTTACTATCT	60	151	XM_001927997.5
	R: TATGCCGTCACAGCGATGAA			
PLIN5	F: CACTCTGTCCTACCCACAGG	60	113	XM_021076783.1
	R: CTGCACTGCGTTCTGCTGG			
PLIN4	F: ATAAGGCCAGGGAGCTGAGA	60	162	XM_021084054.1
	R: AGAAGCTGCTCAGGGTCTTG			
PARP9	F: GCTTCCTTTGTAGGCGGGTG	60	254	XM_021070264.1
	R: ACAGCAGCCCCTTTCTGG			
GAPDH	F: GGCAAATTCAACGGCACAGTCAAG	60	81	NM_001256799.3
	R: TCGCTCCTGGAAGATGGTGATGG			

**Table 2:** Sequencing basic data for three different developmental stages of *Longissimus Dorsi* muscle tissues in Ningxiang pigs.

Terms	Raw Reads Number	Clean Reads Number	Clean Reads Rate	Clean Q30 Bases Rate	Mapped Reads	Mapping Rate
Day 1-1	47582910	46885968	98.53%	90.99%	48173005	95.58%
Day 1-2	45499772	44813742	98.49%	91.02%	45051239	95.35%
Day 1-3	46522890	45826386	98.50%	90.99%	46504851	95.51%
Day 60-1	47532094	46830986	98.53%	91.41%	50456613	95.98%
Day 60-2	44687256	44022350	98.51%	90.69%	47205558	95.68%
Day 60-3	40913650	40292170	98.48%	90.52%	44041267	95.72%
Day 210-1	44536574	44217290	99.28%	92.30%	46642037	95.85%
Day 210-2	45793204	45430838	99.21%	92.14%	47663400	95.53%
Day 210-3	45371844	45096524	99.39%	93.08%	47805991	96.03%

The values represent the reads and proportions that were compared to those in the Ningxiang pig reference genome (Sus scrofa 11.1).

**Table 3:** Muscle development-related candidate genes identified in GO analysis.

Gene	Enriched Pathway	Function	Reference
*RIPOR2*	Regulation of myoblast differentiation; muscle cell development; and skeletal muscle tissue development.	An important factor for myogenic cell fusion and differentiation.	[14]
*MEGF10*	Muscle cell development; myoblast differentiation; and skeletal muscle tissue development.	MEGF10 deficiency impairs skeletal muscle stem cell migration and muscle regeneration.	[15]
*KLHL40*	Skeletal muscle tissue development and muscle cell development.	As a striated-muscle-specific protein, it plays a key role in muscle development and function.	[16]
*PLEC*	Myoblast differentiation; muscle cell development; and skeletal muscle tissue development.	PLEC deficiency leads to a disorganization of myofibrils and sarcomeres.	[17]
*TBX3*	Regulation of myoblast differentiation; myoblast differentiation; and muscle cell development.	TBX3, an essential regulatory factor for muscle and its accessory tissues, plays an important role in muscle and skeletal development.	[18,19]
*FBP2*	Metabolic pathways and glycolysis/gluconeogenesis.	FBP2, besides being a regulatory enzyme of glyconeogenesis, also protects mitochondria against calcium stress and plays a key role in the regulation of the cell cycle.	[20]
*HOMER1*	Skeletal muscle tissue development and muscle cell development.	HOMER1 may influence skeletal muscle function via Ca^2+^.	[21,22]

## Data Availability

The data presented in this study are available on request from the corresponding author.

## Supplementary Materials

The following supporting information can be downloaded at: https://www.mdpi.com/article/10.3390/genes14051050/s1, Table S1. 441 shared DEGs were identified by two comparison groups (day 1 vs. day 60 and day 60 vs. day 210).
